# Diffusion capacity and CT measures of emphysema and airway wall thickness – relation to arterial oxygen tension in COPD patients

**DOI:** 10.3402/ecrj.v3.29141

**Published:** 2016-05-12

**Authors:** Eirunn Waatevik Saure, Per Sigvald Bakke, Tomas Mikal Lind Eagan, Marianne Aanerud, Robert Leroy Jensen, Thomas Blix Grydeland, Ane Johannessen, Roy Miodini Nilsen, Einar Thorsen, Jon Andrew Hardie

**Affiliations:** 1 Department of Clinical Science, Pulmonary Division, University of Bergen, Bergen, Norway; 2 Department of Thoracic Medicine, Haukeland University Hospital, Bergen, Norway; 3 Department of Heart Disease, Haukeland University Hospital, Bergen, Norway; 4 Internal Medicine, Pulmonary Division, University of Utah, Salt Lake City, Utah, USA; 5 Centre for Clinical Research, Haukeland University Hospital, Bergen, Norway

**Keywords:** arterial oxygen tension, diffusion capacity, emphysema, airway wall thickness, computed tomography, COPD

## Abstract

**Background:**

Decreased diffusing capacity of the lung for carbon monoxide (DLCO) is associated with emphysema. DLCO is also related to decreased arterial oxygen tension (PaO_2_), but there are limited data on associations between PaO_2_ and computed tomography (CT) derived measures of emphysema and airway wall thickness.

**Objective:**

To examine whether CT measures of emphysema and airway wall thickness are associated with level of arterial oxygen tension beyond that provided by measurements of diffusion capacity and spirometry.

**Methods:**

The study sample consisted of 271 smoking or ex-smoking COPD patients from the Bergen COPD Cohort Study examined in 2007–2008. Emphysema was assessed as percent of low-attenuation areas<−950 Hounsfield units (%LAA), and airway wall thickness as standardised measure at an internal perimeter of 10 mm (AWT-Pi10). Multiple linear regression models were fitted with PaO_2_ as the outcome variable, and %LAA, AWT-Pi10, DLCO and carbon monoxide transfer coefficient (KCO) as main explanatory variables. The models were adjusted for sex, age, smoking status, and haemoglobin concentration, as well as forced expiratory volume in one second (FEV_1_).

**Results:**

Sixty two per cent of the subjects were men, mean (SD) age was 64 (7) years, mean (SD) FEV_1_ in percent predicted was 50 (15)%, and mean PaO_2_ (SD) was 9.3 (1.1) kPa. The adjusted regression coefficient (CI) for PaO_2_ was –0.32 (−0.04–(−0.019)) per 10% increase in %LAA (*p*<0.01). When diffusion capacity and FEV_1_ were added to the model, respectively, the association lost its statistical significance. No relationship between airway wall thickness and PaO_2_ was found.

**Conclusion:**

CT assessment of airway wall thickness is not associated with arterial oxygen tension in COPD patients. Emphysema score measured by chest CT, is related to decreased PaO_2_, but cannot replace measurements of diffusion capacity in the clinical evaluation of hypoxaemia.

Patients suffering from chronic obstructive pulmonary disease (COPD) are mostly assessed through their history of respiratory symptoms and exacerbations, clinical examination, and spirometry (1). More advanced assessments include measurements of diffusing capacity of the lungs for carbon monoxide (DLCO) and arterial blood gases (ABGs). Computed tomography (CT) scans are not required for the diagnosis of COPD (1) but are widely used for differential diagnosis, that is, pulmonary embolism, lung cancer, bronchiectasis, tuberculosis, interstitial lung disease etc. Further, chest CT is used to determine the distribution of emphysema when surgical intervention is considered. CT derived measures of emphysema and airway wall thickness are related to respiratory symptoms (2) and quality of life (QoL) (3), and predict mortality independently of lung function given by forced expiratory volume in one second (FEV_1_) (4).

However, more data also presents the clinician with a problem of placing the increasing amount of information into context, and to relate results from the various measurements to each other. Low DLCO is associated with a low PaO_2_ (5), and DLCO gives an index of the severity of tissue destruction in emphysema (6, 7). CT derived emphysema score is related to the level pulmonary artery tension in subjects with severe disease (7), but surprisingly, no relationship has been found between CT assessed extent of emphysema and ABG tension (8). To our knowledge, there are no studies examining the association between emphysema score, DLCO and arterial oxygen tension. Furthermore, there are no previous data available on the relationship between airway wall thickness and hypoxaemia.

The aim of this study was to explore the relationship between quantitative measures derived from chest CT scans and ABGs, while controlling for demographics, and pulmonary function tests in a large cohort of well-characterised COPD patients. Understanding patterns of functional impairment and their association to pathological anatomy provides us with one more piece of the COPD puzzle. This could optimise the diagnostic potential and increase the clinical benefit of chest CT scans. We hypothesised that CT-derived quantitative measures of emphysema and airway wall thickness would be associated with lower PaO_2_.

## Methods

The study sample was based on the Bergen COPD Cohort Study (BCCS). The sampling and characteristics of the cohort have been presented in detail elsewhere (9). Briefly, the cohort comprises 433 COPD patients that were recruited in 2006–2007. The main inclusion criteria were a clinical diagnosis age above 40 years, post-bronchodilator FEV_1_/forced vital capacity (FVC) ratio below 0.7, and smoking consumption of at least 10 pack-years (9).

Cross-sectional data on ABGs, chest CT, spirometry, and diffusion capacity were collected at the 1-year follow-up. Of the subjects included at baseline, 395 (91%) were followed up after 1 year. ABGs were collected in 349 subjects, CT scans were collected in 369 subjects, and diffusion measurements were performed in 312 subjects. Overall, 271 participants (63% of the baseline population) had follow-up measurements for ABGs, CT scans, spirometry, and diffusion capacity. There were no large differences between the 271 subjects and the overall study population in terms of sex, age, smoking status, spirometry, height, weight, and level of oxygen tension at baseline (Supplementary Table 1).

### Ethics

The Western Norway Regional Research Ethics Committee approved the study (case number 165.08), and informed consent was obtained from all subjects prior to inclusion.

### ABG measurements

Arterial puncture was done with the subjects sitting upright and at rest for at least 15 min. Samples were analysed on a Radiometer ABL 520 (Radiometer, Copenhagen). In patients who were on long-term oxygen treatment (LTOT), supplemental oxygen was discontinued 30 min prior to drawing the blood sample. In cases where this could not be done due to medical reasons, the samples were excluded from the analyses. The technical procedure for collection and analysis of the ABGs has been described previously (10). Altogether, 46 subjects did not have ABG measurements at the 1-year follow-up for various reasons, including willingness to have arterial blood sampled, supplemental oxygen use, and technical failures.

### Lung function measurements

Subjects underwent spirometry according to American Thoracic Society standards (11) using a Viasys Masterscope (Viasys, Hoechberg, Germany) after bronchodilation with 400 µg of salbutamol. Local reference values for FEV_1_ and FVC were used (12).

Single-breath diffusing capacity of carbon monoxide (transfer factor, DLCO) was measured with a Viasys Healthcare Vmax Encore 22D (Carefusion Viasys Healthcare, Yorba Linda, CA, USA), using a gas mixture of carbon monoxide (0.3%), methane (0.3%), acetylene (0.3%), oxygen (21%), and balance nitrogen. Up to four manoeuvres were performed in order to get two error-free tests. The equipment was calibrated twice daily with a manual calibration syringe, on a weekly basis with biological controls. Also, before each diffusing capacity measure, the device performed an automatic gas calibration for carbon monoxide and methane.

Estimated alveolar volume (VA) was measured from the single-breath dilution of methane, and the diffusion coefficient (KCO) was estimated dividing DLCO by VA. Reference values were calculated using equations from Gulsvik et al. (13).

### Computed tomography

CT scans of the chest were acquired using GE Healthcare multidetector-row CT scanner and obtained in supine position, at suspended full inspiration without administration of intravenous contrast. Exposure settings were 120 kVp and 40 mAs, and images were reconstructed at 1.25 mm contiguous slices, using a low spatial–frequency reconstruction algorithm (GE: Standard). All CT scans were analysed using Pulmonary Workstation 2.0 software (VIDA Diagnostics, Iowa City, IA, USA). Briefly, the lungs were segmented from the thorax wall, heart, and main pulmonary vessels, followed by the segmentation of the individual lobes. The airways were segmented using a region-growing algorithm starting in the trachea and projecting to the smallest airway visible on the CT scan (14). For this study, only third (‘segmental’) to fifth generation airways were analysed, since the number of segmented airways dropped massively beyond the fifth generation.

To avoid potential bias from different distribution of airway sizes between subjects, a standardised measure for airway wall thickness (AWT-Pi10) was derived for each subject by plotting the square root of the airway wall area against the internal perimeter of each measured airway (15). The resulting regression line was used to calculate the square root of the wall area for a ‘theoretical airway’ with an internal perimeter of 10 mm (AWT-Pi10) (15).

The extent of emphysema (% low attenuation areas,% LAA) was estimated using the threshold technique, quantifying the percent of lung voxels with an apparent X-ray attenuation value below −950 Hounsfield units (HU) (16).

### Statistical analyses

Continuous variables were described with mean and standard deviation (SD) when normally distributed, and with median and interquartile range (IQR) when having skewed distributions. Categorical variables were described with number and percentage. Independent samples’ *t*-tests, Wilcoxon rank-sum tests, and one-way analysis of variance (ANOVA) were used to compare continuous variables, and Pearson's chi-squared tests for categorical variables. The descriptive analysis was stratified by sex.

We performed preliminary analyses assessing mean PaO_2_ by tertiles of %LAA, AWT-Pi10, DLCO, and KCO. We used multiple linear regression analyses to examine further associations between PaO_2_ and the explanatory variables %LAA, AWT-Pi10, and diffusion capacity (DLCO and KCO) as continuous variables both in separate models and with all variables in the same model. Adjustments were made for sex, age, smoking status (current/ex), height, and weight (adjustment group 1). To explore if LAA, AWT-Pi10 and/or diffusion capacity was associated with low PaO_2_ beyond the known effect of airflow limitation given by FEV_1_ (10, 17), we added FEV_1_ as a covariate in a separate adjustment group (adjustment group 2). For models with DLCO and KCO, haemoglobin was added as a covariate. We predicted the residuals from the adjusted models. Residual histograms for each of the four models (explanatory variable %LAA, AWT-Pi10, DLCO and KCO, respectively) showed satisfactory normal distribution. The presence of collinearity between predictive parameters representing diffusion capacity and level of emphysema could potentially be a problem in the regression model where both these parameters were present. Therefore, we used variance inflation factor (VIF) to test for multicollinearity in this regression model. Our analyses showed that VIF ranged from 1,23 to 2,32, indicating only very moderate collinearity (Supplementary Table 2). Also, we tested for potential interactions between %LAA, AWT-Pi10, DLCO and KCO, and FEV_1_ and sex. However, none of the interactions tested were statistically significant. For all associations, a significance level of *p*≤0.05 was applied.

All statistical analyses were performed using Stata/SE 11.2 (StataCorp LP, College Station, TX, USA) software for Macintosh.

## Results

The mean (SD) age of the 271 participants was 64 (7) years, and approximately one-third of the sample was women. About 40% of both male and female subjects were current smokers (Table 1). Their mean (SD) post bronchodilator FEV_1_ was 50% (15) of predicted. The mean level of PaO_2_ was significantly lower in women than in men (*p*=0.03), and the frequency of COPD patients with hypoxaemia (defined as PaO_2_ <8 kPa) was approximately twice as high among women than in men (Table 1).

**Table 1:** Characteristics of study participants (*n*=271)

Variable	Total	Female	Male	*p*
Age, mean years (SD)	64.2 (6.7)	63.2 (6.4)	64.8 (6.9)	0.06
Current smoking status, *n* (%)^a^	112 (41)	37 (36)	75 (45)	0.18
Height, mean cm (SD)	171.6 (8.4)	164.1 (5.8)	176.2 (6.3)	<0.01
Weight, mean kg (SD)	75.1 (17.4)	68.6 (17.3)	79.0 (16.3)	<0.01
BMI, mean kg/m^2^ (SD)	25.4 (5.3)	25.4 (5.9)	25.4 (4.9)	0.98
PaO_2_, mean kPa (SD)	9.36 (1.13)	9.17 (1.14)	9.48 (1.11)	0.03
PaCO_2_, mean kPa (SD)	5.26 (0.48)	5.21 (0.49)	5.28 (0.47)	0.23
Hypoxaemia status, n (%)	33 (12.2)	20 (19.6)	13 (7.7)	<0.01
Haemoglobin, mean g/dl (SD)^b^	14.4 (1.2)	13.9 (1.1)	14.8 (1.1)	<0.01
FEV_1_, mean % pred (SD)	50 (14.6)	53.2 (13.5)	48.1 (15.0)	<0.01
FEV/FVC-ratio, median (IQR)	0.46 (0.37–0.55)	0.49 (0.40–0.58)	0.44 (0.35–0.51)	<0.01
GOLD category, n (%)				
II	134 (49)	58 (57)	76 (45)	0.02
III	110 (41)	40 (39)	70 (41)	
IV	27 (10)	4 (4)	23 (14)	
DLCO, mean (SD)	5.16 (1.92)	4.52 (1.58)	5.55 (2.01)	<0.01
DLCO, mean % pred. (SD)	58.4 (18.7)	61.5 (19.3)	56.6 (18.1)	0.04
VA, mean litres (SD)	4.94 (1.07)	4.1 (0.63)	5.5 (0.93)	<0.01
KCO (DLCO/VA), mean (SD)	1.04 (0.31)	1.10 (0.31)	1.01 (0.30)	0.01
LAA both lungs, median (IQR)	13.3 (5.2–19.3)	10.7 (3.9–14.8)	14.9 (6.2–21.8)	<0.01
AWT–Pi10, mean (SD)^b^	4.36 (0.11)	4.31 (0.12)	4.38 (0.10)	<0.01
Total, *n* (%)	271	102 (38)	169 (62)	

a *p*-values are from two-group mean comparison tests (unpaired *t*-tests) and Wilcoxon rank-sum tests for continuous variables and Pearson's chi-squared tests for categorical variables.

b Missing information for current smoking status and AWT–Pi10 (*n*=1) and for haemoglobin (*n*=2).

PaO_2_, Partial pressure of arterial oxygen; PaCO_2_, Partial pressure of arterial carbon dioxide; BMI, body mass index; FEV_1_, Forced expiratory volume in 1 second; FVC, Forced vital capacity; GOLD, Global Initiative for Chronic Obstructive Lung Disease; DLCO, diffusing capacity of the lung for carbon monoxide; KCO, carbon monoxide transfer coefficient; VA, Alveolar volume; %LAA, percentage low attenuation areas; AWT–Pi10, airway wall thickness at an internal perimeter of 10 mm; SD, standard deviation; IQR, inter quartile range.

The mean arterial oxygen tension by tertiles of %LAA, AWT-Pi10, DLCO, and KCO are given in Table 2. The level of arterial oxygen tension decreased with the increasing level of emphysema in terms of %LAA. No trend in level of oxygen tension was observed with increasing level of AWT-Pi10, nor did the level of oxygen tension differs between the three AWT-Pi10 groups. The oxygen tension increased with the increasing level of gas diffusion capacity as measured by DLCO, also after adjusting for the alveolar volume, as given by KCO (Table 2). The increase in oxygen tension showed approximately the same degree of increase across the emphysema tertiles as across the DLCO and KCO tertiles (Table 2).

**Table 2:** Mean (SD) PaO_2_ by tertiles of %LAA, AWT–Pi10, DLCO, and KCO

	PaO_2_ (kPa)
All subjects	9.34 (1.13)
Groups by tertiles of LAA	
I (0.27–6.50)	9.65 (1.14)
II (6.50–15.61)	9.30 (1.18)
III (15.61–53.11)	9.02 (1.05)
*p*-value (one-way ANOVA)	**<0.001**
*p*-value (NP trend)^a^	**<0.001**
Groups by tertiles of AWT-Pi10	
I (3.95–4.30)	9.31 (0.93)
II (4.30–4.40)	9.35 (1.17)
III (4.40–4.87)	9.28 (1.29)
*p*-value (one-way ANOVA)	0.911
*p*-value (NP trend)	0.904
Groups by tertiles of DLCO	
I (1.80–4.18)	8.97 (1.05)
II (4.18–5.61)	9.39 (1.11)
III (5.61–13.91)	9.76 (1.10)
*p*-value (one-way ANOVA)	**<0.001**
*p-*value (NP trend)	**<0.001**
Groups by tertiles of KCO	
I (0.35–0.89)	9.06 (1.04)
II (0.89–1.16)	9.37 (1.16)
III (1.16–2.15)	9.71 (1.10)
*p-*value (One-way ANOVA)	**<0.001**
*p-*value (NP trend)	**<0.001**

Significant *p*-values are indicated in **bold**

a NP trend: nonparametric test for trend across ordered groups.

%LAA, percentage low attenuation areas; AWT-Pi10, airway wall thickness at an internal perimeter of 10 mm; DLCO, diffusing capacity of the lung for carbon monoxide; KCO, carbon monoxide transfer coefficient; SD, standard deviation.

In linear regression analyses with %LAA, AWT–Pi10, DLCO, and KCO as independent variables in four separate models, the %LAA–PaO_2_ relationship remained significant (Table 3), also after adjusting for sex, age, and smoking status. When adding FEV_1_ to the model with %LAA, %LAA was no longer significant associated with PaO_2_. The DLCO–PaO_2_ and the KCO–PaO_2_ relationships remained significant in models adjusting for sex, age, smoking status, and haemoglobin, also when adding FEV_1_ to the model (Table 3). When %LAA and diffusion capacity were entered into the same model, diffusion capacity (given by DLCO or KCO) remained significantly associated with PaO_2_, while %LAA did not (Table 4). The relationship between AWT–Pi10 and PaO_2_ was non-significant.

**Table 3:** Multiple linear regression with %LAA, AWT-Pi10, and diffusion capacity (DLCO or KCO) in four separate models

		Outcome variable PaO_2_ (kPa)		
		
		Unadjusted	Adjustment group 1	Adjustment group 2
Model	Explanatory variable	Coefficient (CI) *p*	Coefficient (CI) *p*	Coefficient (CI) *p*
1	%LAA	**−0.023** **(−0.038–(−0.014))** ***p*****<0.001**	**−0.032** **(−0.044–(−0.019))** ***p*****<0.001**	−0.009 (−0.024–0.006) *p*=0.215
2	AWT-Pi10	−0.174 (−1.27–0.92) *p*=0.76	−0.63 (−1.78–0.51) *p*=0.279	−0.751 (−1.82–0.32) *p*=0.167
3	DLCO	**0.206** **(0.14–0.27)** ***p*****<0.001**	**0.220** **(0.15–0.29)** ***p*****<0.001**	**0.149** **(0.07–0.23)** ***p*****=0.001**
4	KCO	**0.989** **(0.57–1.49)** ***p*****<0.001**	**1.06** **(0.640–1.49)** ***p*****<0.001**	**0.694** **(0.251–1.13)** ***p*****=0.002**

Adjustment group 1=adjusted for sex, age, smoking status, and haemoglobin (in analyses with DLCO and KCO).

Adjustment group 2=adjusted for sex, age, smoking status, haemoglobin (in analyses with DLCO and KCO), and FEV_1_.

Significant results are indicated in **bold**.

%LAA, percentage low attenuation areas; AWT-Pi10, airway wall thickness at an internal perimeter of 10 mm; DLCO, diffusing capacity of the lung for carbon monoxide; KCO, carbon monoxide transfer coefficient; CI confidence interval.

**Table 4:** Multiple linear regression with %LAA and diffusion capacity (DLCO or KCO) in the same model

	Outcome variable PaO_2_ (kPa)	
	
	Adjustment group 1	Adjustment group 2
Explanatory variables	Coefficient (CI) *p*	Coefficient (CI) *p*
%LAA	−0.006 (−0.02–0.01) *p*=0.446	0.004 (−0.01–0.02) *p*=0.651
DLCO	**0.200** **(0.10–0.30)** ***p*****<0.001**	**0.166** **(0.07–0.26)** ***p*****=0.001**

%LAA	−0.013 (−0.03–0.005) *p*=0.151	0.008 (−0.01–0.03) *p*=0.458
KCO	**0.777** **(0.19–1.36)** ***p*****=0.010**	**0.847** **(0.28–1.42)** ***p*****=0.004**

Adjustment group 1=adjusted for sex, age, smoking status, and haemoglobin (in analyses with DLCO and KCO).

Adjustment group 2=adjusted for sex, age, smoking status, haemoglobin (in analyses with DLCO and KCO), and FEV_1_.

Significant results are indicated in **bold**.

%LAA, percentage low attenuation areas; DLCO, diffusing capacity of the lung for carbon monoxide; KCO, carbon monoxide transfer coefficient; CI, confidence interval.

Additional analyses were performed to test whether the emphysema–PaO_2_ relationship were driven by those with severe emphysema. However, after excluding those %LAA>10%, a significant emphysema–PaO_2_ relationship remained.

## Discussion

The main findings of this study were: 1) Low oxygen tension in subjects with COPD was associated with a high level of emphysema, while PaO_2_ was not related to the degree of airway wall thickness measured with chest CT scans; 2) low oxygen tension was associated with low diffusion capacity; 3) degree of emphysema accessed by chest CT scan did not give additional explanation to the level of oxygen tension beyond that provided by diffusion capacity and FEV_1_. In contrast to an earlier study by Biernacki et al. (8), we found a significant association between CT-derived emphysema score and oxygen tension. The finding that the significant emphysema–oxygen tension relationship disappeared after adding DLCO to the equation indicates that the relationship between increasing emphysema and hypoxaemia works mainly through impaired diffusion capacity through loss of surface area for gas exchange and V/Q mismatching.

It has previously been established that an increased ventilation–perfusion (V/Q) mismatch is the main contributor to hypoxaemia in COPD patients (18, 19). In emphysema, a high V/Q ratio, caused by increased physiological dead space due to the destruction of the pulmonary capillary bed, is predominant (20) **. In a study of 14 patients with severe or very severe COPD, V/Q mismatch was found to be the most important cause of hypoxaemia through ‘wasted’ ventilation of poorly perfused areas (21). Also, low V/Q ratios can be present in emphysema. Increasing airflow limitation and closing volume result in alveolar hypoventilation and perfusion of underventilated areas, increasing the V/Q mismatch further (22). When adjusting for either FEV_1_ or diffusion capacity (DLCO or KCO) in our analyses, the relationship between emphysema and PaO_2_ was no longer significant. When including both FEV1 and diffusion capacity in the same model, there was still a significant relationship between diffusion capacity and PaO_2_. This suggests that the emphysema–PaO_2_ association works through both reduced diffusion capacity and airway obstruction. Thus, hypoxaemia in COPD may be better explained by FEV_1_ and diffusion capacity measurements than by CT measurements.

To our knowledge this is the first study to examine the relationship between oxygen tension and measurements of airway wall thickness derived from the chest CT scans. In other studies increased airway wall thickness has been associated with airflow obstruction (23, 24), increased level of dyspnea (15), and impaired quality of life in COPD patients (3, 25). Kim et al. (26) and Mair et al. (27) showed that increased airway wall thickness was associated with the clinical phenotype of chronic bronchitis. We did not find a relationship between airway wall thickness and level of PaO_2_. Airway wall thickness in this study is derived from proximal (third to fifth generation) airways, but it is likely that the airway wall thickness measured also reflects the thickness of more distal airways (28). Because increased airway wall thickness is associated with airway obstruction, one could hypothesise that this would lead to regional hypoventilation, and further increased V/Q mismatch and hypoxaemia. On the other hand, increased airway wall thickness contributes to increased airway wall stiffness that could prevent airway collapse and obstruction.

The present study is a large single-centre study with a vast number of collected data, which allows for extensive adjustment for important confounders. The study represents a unique opportunity to describe the relationships between ABGs, pulmonary function tests, and patho-anatomical information of the chest CT. Another strength of the study is that all CT scans were performed using the same scanner, and all diffusing capacity measurements and blood gas analyses were performed using the same instruments undergoing strict quality controls during the data collection.

One limitation of the present study is that the extent of emphysema is presented as the percentage of the lung showing low X-ray attenuation. However, level of inspiration during the scan, cardiac congestion, fibrosis, and increased phlegm production will reduce %LAA, while image noise can increase the level of %LAA thereby influencing both the sensitivity and specificity of %LAA as an indicator of emphysema. The CT scans were performed with the subject in supine position, while measurements of pulmonary function were done with the subject sitting upright. Prone posture is known to increase lung volume, and differences in gravitational gradient may affect both perfusion and ventilation (29). The supine lung has a larger tissue density than the lung in the prone posture (30, 31). This may potentially lead to underestimation of emphysema scores and thus affect the comparisons and interpretation of results across techniques. Collinearity between predictors of diffusion capacity and level of emphysema could potentially be a problem in analyses where both these variables were present, affecting the validity of the results. However, analyses with VIF showed that multicollinearity was not a problem.

Chest CT scans are increasingly used in the clinical evaluation and for differential diagnosis of subjects with chronic obstructive lung disease (32). The results of this study imply that the CT-assessed level of emphysema is related to oxygen tension in patients with COPD. However, our findings indicate that the CT-assessed level of emphysema does not add information about the oxygen tension beyond that offered by diffusion capacity and FEV_1_.

In conclusion, in a large cohort of COPD patients with mostly moderate disease, high level of emphysema was related to low oxygen tension, but not after adjusting for diffusion capacity or airflow limitation. Airway wall thickness as assessed by CT was not related to oxygen tension.

## Supplementary Material

Diffusion capacity and CT measures of emphysema and airway wall thickness – relation to arterial oxygen tension in COPD patients
