# Supplementary material for: Diffusion capacity and CT measures of emphysema and airway wall thickness – relation to arterial oxygen tension in COPD patients
Source: Eur Clin Respir J. 2016 May 12;3:10.3402/ecrj.v3.29141. doi: 10.3402/ecrj.v3.29141 (PMC4867045; doi:10.3402/ecrj.v3.29141)
Supplement: Diffusion capacity and CT measures of emphysema and airway wall thickness – relation to arterial oxygen tension in COPD patients [file ECRJ-3-29141-s001.doc]

| **Supplement table 1: Comparison of baseline characteristics in all attendants to visit 1 and attendants to the one year follow-up with ABG-, spirometry, CT- and diffusion capacity measurements** | | |
| --- | --- | --- |
| Variable | Attendants to visit 1,  all COPD subjects  included  n = 433 | Attendants to visit 4,  with ABG-, spirometry-, diffusion capacity- and CT data available  n=271 |
| Gender, men, % | 60 | 62 |
| Age, mean (SD), years | 63.5 (6.9) | 64.2 (6.7) |
| Current smokers, % | 43.9 | 41.5 |
| FEV_1_, % predicted mean (SD) | 48.8 (14.4) | 49.9(14.3) |
| PaO_2_, mean (SD), kPa | 9.32 (1.14) | 9.34 (1.08) |
| PaCO_2_, mean (SD), kPa | 5.35 (0.54) | 5.31 (0.47) |
| Height, mean (SD), m | 1.71 (0.09) | 1.72 (0.08) |
| Weight, mean (SD), kg | 74.1 (18.1) | 75.5 (17.4) |
| BMI, mean (SD), kg/m^2^ | 25.3 (5.4) | 25.5 (5.2) |
| **Abbreviations**: FEV_1_ (Forced expiratory volume in 1 second, PaO_2_ (Partial pressure of arterial oxygen), PaCO_2_ (Partial pressure of arterial carbon dioxide), BMI (Body mass index), SD (standard deviation) | | |
| **Supplement table 2: Collinearity between independent predictor variables**  **in multiple linear regression analyses with both %LAA and diffusion capacity**  (DLCO) in the same model   | Variable | VIF, analyses with DLCO | | --- | --- | | | DLCO | 2,32 | | % LAA | 2,30 | | FEV_1_ | 1,74 | | Sex | 1,63 | | Age | 1,28 | | Smoking status | 1,23 | | Hemoglobin | 1,26 |   **Abbreviations**: %LAA (percentage low attenuation areas), DLCO (diffusing capacity  of the lung for carbon monoxide), KCO (carbon monoxide transfer coefficient),  FEV_1_ (Forced expiratory volume in 1 second, VIF (variance inflation factor) | | |
